# How faithfully do HIV clinicians administer the PHQ-9 depression screening tool in high-volume, low-resource clinics? Results from a depression treatment integration project in Malawi

**DOI:** 10.1017/gmh.2019.22

**Published:** 2019-10-02

**Authors:** Brian W. Pence, Melissa A. Stockton, Steven M. Mphonda, Michael Udedi, Kazione Kulisewa, Bradley N. Gaynes, Mina C. Hosseinipour

**Affiliations:** 1 University of North Carolina at Chapel Hill Gillings School of Global Public Health, Chapel Hill, USA; 2 University of North Carolina Project-Malawi, Lilongwe, Malawi; 3 NCDs & Mental Health Unit, Ministry of Health, Malawi, Lilongwe, Malawi; 4 Malawi College of Medicine, Blantyre, Malawi; 5 University of North Carolina at Chapel Hill School of Medicine, Chapel Hill, USA

**Keywords:** PHQ-9, depression, integration, LMICs, reliability, screening

## Abstract

**Background.:**

Integration of mental health services into nonspecialist settings is expanding in low and middle income countries (LMICs). Among many factors required for success, such programs require reliable administration of mental health screening tools. While several tools have been validated in carefully conducted research studies, few studies have assessed how reliably nonspecialist clinicians administer these tools to low-literacy LMIC populations in routine care.

**Methods.:**

Ninety-seven patients accessing human immunodeficiency virus primary care in Malawi who completed Patient Health Questionnaire (PHQ)-9 depression screening with their clinician then completed a second PHQ-9 with a trained research assistant (RA) blinded to the first result.

**Results.:**

Compared to clinicians, RAs identified more patients with any depressive symptoms (PHQ-9 score ⩾5: 38% *v*. 32%), moderate/severe symptoms (PHQ-9 ⩾ 10: 14% *v*. 6%), any suicidality (14% *v*. 4%), and active suicidality (3% *v*. 2%). Across these indicators, clinician and RA ratings had strong overall agreement (81–97%) but low corrected Kappa agreement (31–59%). Treating RA results as the reference standard of a carefully supervised research administration of the PHQ-9, clinician administration had high specificity (90–99%) but low sensitivity (23–68%) for these indicators.

**Conclusions.:**

In routine care in LMICs, clinicians may administer validated mental health screening tools with varying quality. To ensure quality, integration programs must incorporate appropriate and ongoing training, support, supervision, and monitoring.

## Introduction

Depression is one of the most common comorbidities among people living with human immunodeficiency virus (HIV) and poses substantial barriers to HIV treatment success. People living with HIV who also have depression consistently demonstrate worse antiretroviral (ART) medication adherence, reduced engagement in care, greater likelihood of ART treatment failure, faster disease progression, and higher mortality rates (Pence *et al*. 2007; Gonzalez *et al*. 2011; Nakimuli-Mpungu *et al*. 2011; Franke *et al*. 2013; Smillie *et al*. 2014; Kidia *et al*. 2015). As such, increasing attention is focusing on the importance of integrating depression screening and treatment into routine HIV care (Pence *et al*. 2012).

In many low- and middle-income countries (LMICs), access to mental health professionals is scarce and resources to establish specialty mental health services are largely lacking (World Health Organization, 2008). Therefore, to be successful, efforts to expand mental health care for patients with HIV must focus on integrating mental health screening and treatment into the workflow of existing non-specialist clinic personnel. Such efforts must balance a desire for high-quality care against considerations of feasibility and pragmatism, given the high patient volumes and heavy time demands that are typical of many LMIC public health care settings.

With the goal of expanding access to mental health care, a robust evidence base now supports the effectiveness of ‘task-shifting’ care models, or models that train general clinicians to safely prescribe mental health medications and train lay health workers to provide psychosocial counseling (Singla *et al*. 2017). However, before patients can be treated, they must be identified. Focusing on depression in particular, one barrier to identification and subsequent treatment is the integration of either targeted or universal depression screening into routine care. Little research has examined the quality of depression screening when administered by support personnel or non-specialist health care workers in routine care. In addition, in most LMIC settings illiteracy is high, meaning that screening tools must be administered by a staff person rather than self-administered. Therefore, the published estimates of sensitivity and specificity for depression screening tools from carefully implemented validation studies in the literature may not reflect the tools' performance in leading to correct clinical identification of depression under such real-world conditions.

Accordingly, we undertook a study of the reliability of administration of a standard depression assessment tool by HIV post-test counselors and HIV nurses and clinicians in two public-sector primary health care clinics in Lilongwe, Malawi, participating in a project focused on integrating depression screening and treatment into routine HIV care.

## Methods

The present study was nested within an ongoing study designed to estimate the impact of integrated depression treatment on HIV care outcomes among non-pregnant adult patients newly starting ART (Udedi *et al*. 2018). As part of this study, universal depression screening was integrated into two public-sector primary care ART clinics in Lilongwe, Malawi, from April 2017–November 2018. Depression screening followed a two-stage process. First, all patients receiving a positive HIV test and completing HIV post-test counseling were asked the two-item Patient Health Questionnaire (PHQ)-2 by the HIV post-test counselor. The PHQ-2 asks about the presence within the past two weeks of the two core symptoms of a depressive episode (depressed mood or lack of interest in usual activities) based on the Diagnostic and Statistical Manual (DSM)-5 (American Psychiatric Association, 2013). Patients reporting any experience of either symptom in the past two weeks (PHQ-2 score >0) were given a paper PHQ-9 to give to their ART clinical officer or nurse during their ART intake appointment. The PHQ-9 begins with the two questions from the PHQ-2 (which the clinician repeats) and then asks about the presence of the other seven possible DSM-5 symptoms of a depressive episode. The total PHQ-9 score can range from 0–27, with scores between 0 and 4 typically considered no depression, 5–9 mild depression, 10–14 moderate depression, 15–19 moderately severe depression, and 20–27 severe depression. As a single cutpoint, a score of ⩾10 is typically considered indicative of probable major depression (Spitzer *et al*. 1999). The study protocol further assessed severity of suicidal ideation using an additional suicide risk assessment tool for any participant who endorsed item 9 on the PHQ-9.

The goal of the present substudy was to compare the results of the PHQ-2 and PHQ-9 administered by the HIV post-test counselor and HIV clinician during routine clinical care to a second PHQ-9 administered by a trained study research assistant (RA). For this substudy, patients who completed the depression screening protocol during their regular clinical appointment during an 8-week period (September 19^th^ through November 14^th^, 2018) completed a second PHQ-9 with a RA after the end of their clinical appointment. Thus the two assessments occurred on the same day, typically within the space of a few hours. The RA was blinded to the results of the clinical PHQ-2 and PHQ-9. No mention was made to clinical staff that the substudy was underway so as not to affect clinician behavior. Although varying the sequencing of the two PHQ-9 assessments would have been ideal, it was deemed not feasible to do so without revealing the substudy to clinicians and thereby likely influencing clinician behavior.

For the substudy, RAs completed a one-day training with a mental health researcher which included review of the definition and symptoms of depression, detailed review of the individual PHQ-9 questions, supervised practice administering the PHQ-9 to each other, and finally supervised practice administering the PHQ-9 to patients. Practice with feedback continued until the supervisor was satisfied that the RAs were administering the tool properly. A refresher training occurred at the midpoint of data collection.

Clinicians were general practice clinical officers and nurses who had no training in mental health before the project apart from brief orientation as part of medical or nursing education. At the start of the project, all clinicians were trained in the administration of the PHQ-9 in a half-day training that did not include a practical component. For clinicians, biweekly supervision from a mental health specialist was offered at both clinics that addressed PHQ-9 administration among other topics, although attendance at these supervision sessions was not required and was inconsistent.

Once the research PHQ-9 was completed, the RA compared its results to the PHQ-2 and PHQ-9 completed by the HIV post-test counselor and clinician, and any clinical action already taken by the clinician was reviewed. Patients who were identified as having mild, moderate, or severe depressive symptoms (PHQ-9 score ⩾5) or suicidal thoughts or behaviors on the research PHQ-9 who had not already been identified clinically were assessed for safety and referred to appropriate mental health treatment.

### Statistical analyses

Our purpose in this substudy was to evaluate how reliably clinicians identified patients needing depression treatment or suicidality assessment, relative to trained RAs using the same screening tool. We compared the results from the first two questions of the RAs' PHQ-9, identical to the PHQ-2, to the HIV post-test counselors' PHQ-2 results. Among those with a PHQ-2 score >0, we then compared the number of patients falling in the no, mild, moderate, and severe depression categories, as well as those being identified with no suicidality *v*. passive or active suicidality, between the clinicians' and the RAs' ratings. We present simple concordance tables as well as sensitivity, specificity, overall agreement, and a corrected Kappa statistic comparing clinicians' assessment with that of the RAs.

Our assumption was that our trained and supervised RAs would administer the PHQ-9 reliably. Our hypothesis was that clinicians would tend to under-diagnose depressive symptoms and suicidality relative to the trained RAs. Therefore, we treated the RA assessment as the ‘reference standard,’ and calculated the sensitivity and specificity of clinicians' identification of elevated depressive symptoms relative to RAs' identification. We note that the RA rating does not represent a diagnostic reference standard, but rather a reference standard of the assessment results expected when the PHQ-9 is administered by carefully supervised research staff as is common in most validation studies.

### Human subjects approval

All parent and substudy activities were approved by the National Health Sciences Research Council of Malawi (NHSRC) and the University of North Carolina at Chapel Hill Biomedical Institutional Review Board. All patients provided written informed consent.

## Results

Over a ten-week period, 156 patients newly initiated ART at our study sites. Of these, 149 completed the regular clinical depression assessment; 97 patients (65%) further provided informed consent for this substudy and are included in the analysis (62 from clinic A and 35 from clinic B). Those patients not included (*n*  =  52) for the most part were not approached because of administrative delays in securing cash for transport reimbursements for participants. Participants had a mean age of 33 years (range: 19–56 years) and 59.8% were female, reflecting the demographics of those newly starting ART care (Table 1). All were classified as having HIV infection in WHO stage I (asymptomatic).

**Table 1.** Characteristics of sample

Characteristic	Mean (s.d.)	*n* (%)
Overall		97 (100%)
Clinic		
A		62 (63.9)
B		35 (36.1)
Age	33 (9.1)	
Sex		
Male		39 (40.2)
Female		58 (59.8)
WHO disease stage	
I	97 (100)	

Overall, the RAs identified a higher proportion of patients scoring positive on the initial PHQ-2 (55% *v*. 44%), as having any depressive symptoms (mild, moderate, or severe; 38% *v*. 32%), and as having moderate to severe depressive symptoms (14% *v*. 6%) (Table 2). Twelve patients (13%) were classified as either PHQ-2 screen-negative or not depressed by clinicians but were identified as having at least mild depressive symptoms by RAs; six patients (6%) were not identified by RAs but were identified as having at least mild depressive symptoms by clinicians (Table 3).

**Table 2.** Prevalence of depressive symptoms and suicidal ideation according to RAs’ and HIV providers’ assessments

	RA (%)	Clinician^a^ (%)
Initial screen-positive (PHQ-2 > 0)	55	44
Mild, moderate, or severe depressive symptoms (PHQ-9 ⩾ 5)	38	32
Moderate or severe depressive symptoms (PHQ-9 > 9)	14	6
Any suicidal thoughts	14	4
Active suicidal thoughts	3	2

a For initial screen-positive: Clinician is the HIV post-test counselor. For all other measures, clinician is the HIV nurse or clinical officer.

**Table 3.** Comparison of PHQ-2 and PHQ-9 screening results between HIV testing counselors and clinicians and RA

		RA rating					
HTC counselor/HIV			Screen-positive and depressive symptoms				
Clinician rating		Screen-negative	Absent	Mild	Moderate	Severe	Total
	Screen-negative	39	6	5	4	0	54
Screen-positive & depressive symptoms	Absent	2	7	3	0	0	12
	Mild	3	3	13	5	1	25
	Moderate	0	0	2	2	0	4
	Severe	0	0	0	0	2	2
	Total	44	16	23	11	3	97

Green areas indicate patients whom the provider assessed as having greater severity than the RA.

Blue areas indicate patients whom the provider assessed as having lower severity than the RA.

In assessing suicidality, RAs identified a higher proportion of patients as having any suicidality (14% *v*. 4%) and active suicidality (3% *v*. 2%). Ten patients reported any suicidality to RAs who had not reported any to the clinician, while one patient reported any suicidality to the clinician but did not report it to the RA (Table 4).

**Table 4.** Comparison of assessment of suicidal thoughts between HIV testing counselors and clinicians and RAs

HTC counselor/HIV	RA rating				
Clinician rating	None	Passive	Active, low risk	Active, high risk	Total
None	81	8	2	0	91
Passive	1	1	0	0	2
Active, low risk	0	1	0	0	1
Active, high risk	0	0	0	1	1
Total	82	10	2	1	95^a^

a Excludes those who don't have provider SRAP results (*n*  =  2).

Green areas indicate patients whom the provider assessed as having greater severity than the RA.

Blue areas indicate patients whom the provider assessed as having lower severity than the RA.

When the RAs' PHQ-9 ratings were treated as the reference standard, the clinicians' assessments had 68% sensitivity and 90% specificity in identifying patients with at least mild depressive symptoms, and 29% sensitivity and 98% specificity in identifying patients with moderate to severe depressive symptoms. Overall agreement for at least mild depressive symptoms was 81%, and the corrected Kappa statistic, or the extent to which overall agreement exceeded that which would be expected from chance alone, was 59%. For moderate to severe depressive symptoms, overall agreement was 88% and the corrected Kappa statistic was 34%. In identifying patients with any suicidal thoughts, clinicians' ratings had 23% sensitivity and 99% specificity; overall agreement was 88% and the corrected Kappa was 31%. For active suicidal thoughts, clinicians' ratings had 33% sensitivity and 99% specificity (Table 5).

**Table 5.** Sensitivity and specificity of HIV test counselors and clinicians relative to RAs in identifying depression and suicidal thoughts

	Cases	Non-cases	Sensitivity (%)	Specificity (%)	Overall agreement (%)	Corrected Kappa
Initial screen-positive (PHQ-2 > 0)	53	44	72	89	79	0.592
Mild, moderate, or severe depressive symptoms (PHQ-9 ⩾ 5)	37	60	68	90	81	0.594
Moderate or severe depressive symptoms (PHQ-9 > 9)	14	83	29	98	88	0.343
Any suicidal thoughts^a^	13	82	23	99	88	0.308
Active suicidal thoughts^a^	3	92	33	99	97	0.384

a Excludes those who don't have provider SRAP results (*n*  =  2).

## Discussion

In this study in Malawi, we compared results of screening for depression and suicidal thoughts when conducted by existing clinical staff, as would be done in real-world clinical settings with low-literacy populations, to results of the same screening when conducted by dedicated RAs, as has been done in most validation studies, in both cases using the same validated depression screening instrument (the PHQ-9). We hypothesized that clinicians in routine care would under-identify depressive symptoms and suicidal thoughts relative to dedicated RAs using the same tool. Overall, we found moderate concordance between clinician and RA assessments, but found that clinicians consistently identified fewer cases of depressive symptoms and suicidal thoughts relative to RAs. If RA assessments were treated as the ‘reference standard’ of results expected from careful screening administration, clinicians' assessments had high specificity but moderate to low sensitivity in identifying all depression, moderate to severe depression, any suicidal thoughts, and active suicidal thoughts.

Substantial research has been conducted to validate screening tools for common mental disorders such as depression in many cultures around the globe and in the sub-Saharan Africa region (Gibson *et al*. 2009; Ali *et al*. 2016; Chorwe-Sungani & Chipps, 2017). However, the manner or method of administration of these tools has received relatively little attention. The difference in our results between clinicians in real-world practice and dedicated RAs in a more-controlled research environment suggests that setting and administration may affect the outcomes of depression screening efforts.

A small number of related studies have found similar results. A study in South Africa (Breuer *et al*. 2012) compared administration of the Substance Abuse and Mental Illness Symptoms Screener (Pence *et al*. 2005) between mental health nurses and lay adherence counselors one week later. The study found fair inter-rater reliability but found that lay counselors detected symptoms more frequently than the mental health nurses. A study in Uganda (Akena *et al*. 2016) compared administration of the PHQ-9 between lay health care workers and masters-level psychologist RAs, finding moderate correlation between the two cadres of administrators.

Depression affects a large proportion of people living with HIV and negatively affects HIV care engagement and treatment outcomes. As interventions to increase universal HIV testing and counseling (HTC) and access to early ART continue to expand in LMICs, integration of mental health services into HIV programs will be an important means of ensuring optimal care and treatment outcomes. To effectively reach those in need, such service integration efforts must incorporate routine or targeted depression screening. The present study suggests that such screening tools may perform less reliably when integrated into routine care than when validated in carefully controlled research studies, and that ongoing training, supervision, and support will be necessary to ensure that depression screening functions effectively to identify those in need of services. Of course, screening on its own in the absence of treatment options both is ineffective (O'Connor *et al*. 2009) and raises significant ethical concerns. Screening must be paired with availability of mental health services, potentially drawing on the large literature demonstrating the effectiveness of task-shifted mental health interventions in LMICs (Singla *et al*. 2017).

While the RAs in our study identified more case of depressive symptoms than the existing providers, the providers' PHQ-9 assessments still had a relatively high level of agreement with the RAs' PHQ-9 assessments of any depressive symptoms (81% overall agreement) and moderate to severe depressive symptoms (88% overall agreement). Additionally, there were some cases where the providers identified cases of depression that were not identified by the RAs. These findings may reflect certain patients being more comfortable disclosing symptoms to their provider than to a RA, or may reflect the underlying potential for inter-rater variability in PHQ-9 administration.

Beyond the higher identification of depressive symptoms, a greater number of patients identified any suicidal thoughts and active suicidal thoughts with the RA than with the clinician. It is possible that patients were more open to revealing suicidal thoughts to RAs because of a sense of time pressure during the clinical appointment or for other reasons. Ensuring that suicidal thoughts can be accurately identified and addressed clinically should be a key component of mental health integration efforts.

Many of the common challenges to integrating depression screening and treatment into routine care may also factor into providers' underdiagnoses of depression. In Malawi, as in other LMICs, workload is high in the public health sector, high turnover leads to additional responsibilities above the staff's comfort level (Kakuma *et al*. 2011), and there is an overwhelming shortage of mental health human resources (World Health Organization, 2008). In anticipation of some of these challenges, the parent study designed the administration of the PHQ-9 to be split up such that the HIV testing and counseling (HTC) counselors administer the initial screen with the PHQ-2 during HTC and (for those with a positive result on the PHQ-2) the nurses or clinicians administer the remaining seven questions of the PHQ-9 during ART initiation. The lack of infrastructure and private spaces may also play an important role in underdiagnoses, as clinicians may rush when administering the PHQ or patients may feel uncomfortable to responding to sensitive PHQ-9 questions without confidentiality. All of these factors may have hampered the quality of the providers' PHQ-9 screening.

### Limitations

There are several limitations to this study. First, while we treated the RAs' assessment results as the reference standard, this analysis does not assume that their assessment should be viewed as a gold standard diagnostic evaluation of depression. Rather, their assessments represent the performance of the PHQ-9 when administered by carefully supervised research staff, as is generally done in validation studies, to which clinicians' assessments can be compared. While the PHQ-9 has not been validated specifically among PLHIW in Malawi, it has been validated in Malawi among patients engaged in diabetes care (Udedi *et al.* 2019) and has been validated in many other countries and languages around the world, with strikingly consistent results from context to context (Monahan *et al*. 2009; Gelaye *et al*. 2013; Cholera *et al*. 2014; Levis *et al*. 2019). Further research assuring the validity of depression screening and management tools will support the implementation and effectiveness of depression treatment programs in Malawi and other SSA countries. Finally, systematic bias could have arisen from the order in which patients were screened. While ideally the order of the clinical and research PHQ-9s would have been randomized, it was only possible for the RAs to rescreen patients after they had completed their clinical visits as to not disrupt routine care.

## Conclusions

Human resources for mental health continue to be inadequate in resource-limited settings (World Health Organization, 2008; Patel *et al*. 2018). Task shifting and development of simple and easy to use tools to screen and treat patients has proven to be an effective and feasible approach to providing mental health care (Singla *et al*. 2017). Findings from this study suggest that provider administered depression screening using the PHQ-9 as part of ART care is feasible and does lead to the identification of patients with high-depressive symptoms. However, providers using the PHQ-9 in routine care may under-identify cases of depressive symptoms and suicidal ideation. Thus to maintain fidelity of implementation, integration programs must incorporate appropriate training, support, and supervision as well as monitoring of clinical skills and practice.
